# Lung (extracorporeal CO_2_ removal) and renal (continuous renal replacement therapy) support: the role of ultraprotective strategy in Covid 19 and non-Covid 19 ARDS. A case-control study

**DOI:** 10.1186/s44158-024-00164-4

**Published:** 2024-04-27

**Authors:** Daniela Pasero, Laura Pistidda, Davide Piredda, Corrado Liperi, Andrea Cossu, Raffaella Esposito, Angela Muroni, Cristiano Mereu, Carlino Rum, Gian Pietro Branca, Franco Mulas, Mariangela Puci, Giovanni Sotgiu, Pierpaolo Terragni

**Affiliations:** 1 Anesthesia and General Intensive Care Unit, AOU Sassari, Sassari, Italy; 2 Department of Medicine, Surgery and Pharmacy, University of Sassari, A.O.U Sassari, Viale San Pietro 43, 07100 Sassari, Italy; 3 Clinical Epidemiology and Medical Statistics Unit, University of Sassari, Sassari, Italy

**Keywords:** ARDS, Lung-protective ventilatory strategy, Low-Flow CO2 removal, AKI, CRRT, COVID-19

## Abstract

**Background:**

Preliminary studies suggest that moderate ARDS and acute renal failure might benefit from extracorporeal CO_2_ removal (ECCO_2_R) coupled with CRRT. However, evidence is limited and potential for this coupled treatment may need to be explored. The aim of the present study was to evaluate whether a protective driving pressure was obtained applying low-flow ECCO_2-_R plus CRRT in patients affected by moderate ARDS with COVID-19 compared to an historical group without COVID-19.

**Methods:**

A case-control study has been conducted comparing a group of consecutive moderate ARDS patients presenting AKI and affected by COVID-19, who needed low-flow ECCO_2-_R plus CRRT to achieve an ultra-protective ventilatory strategy, with historical group without COVID-19 that matched for clinical presentation and underwent the same ultra-protective treatment. V_T_ was set at 6 mL/kg predicted body weight then ECCO_2_R was assessed to facilitate ultra-protective low V_T_ ventilation to preserve safe Pplat and low driving pressure.

**Results:**

ECCO_2_R+CRRT reduced the driving pressure from 17 (14-18) to 11.5 (10-15) cmH_2_O (p<0.0004) in the fourteen ARDS patients by decreasing V_T_ from 6.7 ml/kg PBW (6.1-6.9) to 5.1 (4.2-5.6) after 1 hour (*p* <0.0001). In the ARDS patients with COVID-19, the driving pressure reduction was more effective from baseline 18 (14-24) cmH_2_O to 11 (10-15) cmH_2_O (p<0.004), compared to the control group from 15 (13-17) to 12(10-16) cmH_2_O (p< 0.03), after one hour. ECCO_2_R+CRRT did not affected 28 days mortality in the two groups, while we observed a shorter duration of mechanical ventilation (19 {7-29} vs 24 {22-38} days; p=0.24) and ICU length of stay (19 {7-29} vs 24 {22-78} days; p=0.25) in moderate ARDS patients with COVID-19 compared to control group.

**Conclusions:**

In moderate ARDS patients with or without COVID-19 disease, ECCO_2_R+CRRT may be and effective supportive treatment to reach protective values of driving pressure unless severe oxygenation defects arise requiring ECMO therapy initiation.

## Background

Mechanical ventilation (MV) is the main form of life support for patients with acute respiratory failure and can resolve the impairment of gas exchange alteration in the vast majority of patients [1]. Tidal volume (V_T_) limitation to 6 ml/kg predicted body weight (PBW) and Plateau pressure (P_plat_) control to a limit of 30 cm H_2_O represent the standard of care for MV in ARDS patients [2].

As tidal hyperinflation may occur in some patients despite limiting V_T_ and P_plat_, they may benefit from V_T_ reduction even if ventilated less than 30 cm H2O of P_plat_ [3].

Improving technology for acute respiratory failure treatment might involve the low-flow ECCO_2_R system, that facilitates a lower volume (4ml/Kg of PBW) and lower pressure (P_plat_ < 30 cmH_2_O) aiming at reaching an “ultra-protective” MV strategy in patients with moderate-mild ARDS [4]. Critically ill patients undergoing MV often suffer of acute kidney injury (AKI) and frequently (between 35% to 60% of cases) require Continuous Renal Replacement Therapies (CRRT) because of multiorgan failure [5, 6].

Over the past twenty years a large number of experimental attempts have tried to couple ECCO_2_R with CRRT systems into specific lung-renal support, allowing “ultra-protective” settings in ARDS patients affected by AKI [7]. In a recent trial, the use of CRRT plus ECCO_2_R allowed ultra-protective MV resulting in better recovery of renal function, lower concentration of inflammatory mediators and lower plasma pro-apoptotic activity [7].

Even if less explored, the relative contribution of ECCO_2_R on total CO_2_ clearance and renal support on the natural lung became of specific interest during the last pandemic emergency characterized by the great prevalence of severe acute respiratory distress.

In this scenario, which rapidly became a global health emergency, lung damage was not the only presentation of the complex syndrome.

Currently, it is well known that SARS-CoV-2 infection might also involve other organs/systems presenting with extra-respiratory manifestations, including renal, cardiac, gastrointestinal, hepatic, neurological, olfactory, gustatory, ocular, cutaneous and hematological symptoms.

The need to treat multiple organ failure during the emergency period induced clinician to extend the concepts on extracorporeal CO_2_ removal, introduced in 1977 to control arterial CO_2_ tension and reduce ventilation thus allowing lung rest, directly translating these strategies to pathological conditions caused by SARS-CoV-2 infection.

Several extra-respiratory manifestations, such as acute kidney injury, coagulation disorders and thrombotic complications, represented a challenge while supporting lung and kidney.

The aim of the present study was to evaluate whether a protective driving pressure was obtained applying low-flow ECCO_2-_R plus CRRT in patients affected by moderate ARDS with COVID-19 compared to an historical group of patients not affected by COVID-19.

## Methods

### Study design and setting

A case-control study was conducted at the Intensive Care Unit of the University Hospital of Sassari, Italy. All consecutive patients affected by moderate ARDS with COVID-19 and AKI with CRRT who needed an ultraprotective ventilatory strategy were evaluated and underwent a low-flow CO_2_-removal plus CRRT treatment. Instead, an historical group of patients affected by moderate ARDS without COVID-19, who had been given ultraprotective treatment with ECCO_2_R was retrospectively collected as control.

The study was approved by our Institution’s Ethics’ Committee (*ASL 1 Sassari -Prot. 2440/CE*); all methods were performed in accordance with the relevant guidelines and regulations and informed consent to participate was obtained according to Italian regulations.

### Participants sampling and inclusion

The ultraprotective strategy was applied to patients affected by moderate ARDS who developed a respiratory acidosis that could not be managed with the sole low VT because respiratory mechanic was not protective for driving pressure and Pplat and underwent already CRRT because of AKI. The ultraprotective strategy was achieved by applying a membrane lung to a renal replacement circuit in patients who were already on CRRT treatment.

Inclusion criteria were: need for CRRT and MV with concomitant hypercapnic respiratory acidosis in moderate ARDS (PaO2/FiO2 100–200 mmHg, with positive end-expiratory pressure (PEEP) ≥ 5 cmH2O) according to the Berlin definition, patients expected to receive invasive MV for > 24 hours and age > 18 years old. Exclusion criteria were chronic obstructive pulmonary disease, pregnancy, intracranial abnormality, heart insufficiency or acute coronary syndrome and contraindication for systemic anticoagulation.

The primary endpoint was to evaluate whether patients affected by moderate ARDS and COVID-19 could achieve a driving pressure ≤ 14 cmH_2_O applying ultraprotective strategy with ECCO_2_R compared to control group.

The secondary endpoints were: a) days on mechanical ventilation; b) ICU length of stay and c) mortality at 28 days.

### Variables and data measurements

Severity of comorbidities in all patients were categorized through Charlson Index. To assess organ failure and to predict mortality, SOFA score plus Simplified Acute Physiological Score (SAPS II) and Acute Physiologic Assessment and Chronic Health Evaluation II (APACHE score II) were assessed.

Patients enrolled were sedated, paralyzed, and ventilated. Neuromuscular blockade was administered for a minimum of 24 hours. V_T_ was set at 6 mL/kg PBW and PEEP was adjusted to obtain a P_plat_ between 28 and 30 cmH2O.

ECCO_2_R was assessed to facilitate ultra-protected low V_T_ ventilation in order to preserve safe P_plat_ values and low Driving Pressure [3, 4, 8].

V_T_ was progressively reduced towards the value of 4 mL/kg PBW and PEEP titrated to a target P_plat_ of 23–25 cmH_2_O. Sweep gas and blood flow were set to maintain a pH >7.28. If PaCO_2_ exceeded 75 mmHg and/or despite optimal ECCO_2_R settings and a respiratory rate (RR) of 30-35 breaths/min, V_T_ was increased to the last previously tolerated value. In case CO_2_ and pH control were achieved with the goal of ultra-protected low V_T_ ventilation, RR was progressively reduced.

The potential for weaning off ultra-protective low V_T_ ventilation was assessed after 72 hours of ECCO_2_R treatment by setting MV according to the conventional ARDS*Net* strategy (increasing V_T_ to 6 ml/kg PBW with RR = 20–30 breaths/min and applying PEEP-FIO_2_ combination setting, then switching off the sweep-gas flow through the ECCO_2_R device). If P_plat_ remained at less than 28 cm H_2_O, ECCO_2_R was stopped and conventional ARDS*Net* ventilatory strategy reestablished. CRRT was continued or suspended depending on the recovery of renal function regardless of respiratory management [4].

Respiratory mechanics, hemodynamic parameters, arterial blood-gas values, heparin dose, and activated partial thromboplastin time ratio (APTT*ratio*) were collected during extracorporeal support at baseline and run-in-time after 24, 48 and 72 hours. Blood-chemistry determinations were obtained daily. Respiratory-system mechanics data were calculated according to standard formulas. Hemodynamic parameters and temperature were monitored continuously; diuresis and arterial blood gases were measured during the whole stay at the intensive care unit (ICU). Norepinephrine infusion was applied to fluid administration to reach a target mean arterial pressure of 65 mmHg.

For extracorporeal treatment, a 13-Fr hemodialysis venous catheter (GamcathTM^®^; Gambro-Baxter), under ultrasonography guidance, was percutaneously inserted into the femoral vein.

ECCO_2_R was provided by a low-flow CO_2_-removal device with concomitant CRRT in CVVHDF setting, with a polymethylpentene hollow fiber gas-exchanger membrane (Prisma Lung+^®^ or OMNI^®^ platform). CRRT was performed through dialysis membranes ST150^®^ Gambro-Baxter or OMNI filter^®^ dialysis-Bbraun. The venous blood was actively convoyed via roller pump with an up to 450 ml/min individual-adapted flow to the exchanger membrane. CO_2_ removal was reached by diffusion with 10 L/m O_2_ gas flow. During treatment, a cleaning of oxygenator fibers was performed by increasing for a few seconds O_2_ gas flow. Anticoagulation was performed through systemic heparin infusion targeting an aPTT*ratio* range of 1.8-2.2 [4].

## Sample size

In a favorable scenario with low driving pressure variability, a sample size of 14 produces a two-sided 95% confidence interval with a distance from the mean to the limits of 0.524 when the known standard deviation is 1; however, in the worst scenario described in the literature, a sample size of 14 produces a two-sided 95% confidence interval with a distance from the mean to the bounds of 1.571 when the known standard deviation is 3 [9, 10].

## Statistical analysis

Patients enrolled with clinical pneumonia due to SARS-CoV-2 were submitted to analysis independently from patients with other causes of pneumonia (historical group of non-COVID ARDS patients).

Sample characteristics were summarized as median and 25^th^-75^th^ percentiles (IQR) for quantitative variables, and by absolute and relative (percentage) frequencies for qualitative variables. The comparison between COVID-19 patients was carried out using Fisher exact and Mann-Whitney tests, as appropriate. Differences among study time-points were analyzed using Wilcoxon signed-rank test or non-parametric analysis of variance for repeated measures (Friedman test). In case of a significant Friedman test, for post hoc multiple testing, we adjusted the alpha level by using the Bonferroni correction (i.e., results were considered statistically significant at p <0.005). For all other analyses, a p-value lower than 0.05 was considered statistically significant. STATA17 statistical software was used for statistical computation.

## Results

Between April 2018 and November 2022, 14 adult patients with moderate ARDS (the group affected by COVID-19 disease and the control group of non-COVID ARDS patients from the prepandemic period) were admitted to ICU at the University Hospital in Sassari, Italy.

At baseline, the median (*IQR)* of PaO2/FiO2 ratio in COVID-positive and COVID-negative patients was 107 (95-130) and 153 (102-202), respectively (*p-value* 0.25); overall, a PaO2/FiO2 ratio of 121.5 (102-155) was observed. All patients were assisted in volume-controlled mode ventilation and received a propofol and opiate-based on sedation and pain relief regime; they were evaluated as class III according to the Kidney Disease Improving Global Outcomes (KDIGO) and were already undergoing continuous veno-venous hemodialysis (CVVHD) when the CO_2_ hollow filter was applied, because of acute renal failure and oliguria.

Comparison of demographic data, respiratory variables, causes of lung injury, adjuvant therapy before ECCO_2_R and clinical characteristics stratified by COVID-19 patients are shown in Table 1.

**Table 1:** Comparison of demographic and clinical characteristics stratified by COVID-19 patients

**Variables**^**a**^	**overall (*n*= 14)**	**COVID-19 negative (*n*= 7)**	**COVID-19 positive (*n*= 7)**	***p*-value**
Age,* years*	65 (62-72)	62 (62-69)	72 (60-74)	0.55
Males, n (%)	9 (64.3)	4 (57.1)	5 (71.4)	1.00
BMI, *kg/m*^*2*^	28.4 (25.6-29.4)	27.5 (25.6-29.4)	29.3 (24.5-29.4)	0.83
APACHE II	14.5 (10-20)	19 (10-22)	10 (10-18)	0.19
SAPS II	37 (34-50)	45 (34-55)	36 (34-38)	0.33
Charlson Index	3 (2-6)	3 (2-4)	5 (2-6)	0.64
**SOFA parameters at baseline**				
SOFA total score	8.5 (6-9)	9 (6-11)	8 (5-9)	0.24
GSC	14.5 (14-15)	14 (7-15)	15 (14-15)	0.48
PaO2/ FiO2* ratio*	121.5 (102-155)	153 (102-202)	107 (95-130)	0.25
Mean arterial pressure,* mm Hg*	73.5 (68-80)	75 (65-80)	72 (70-75)	0.88
Creatine kinase*, mg/dL*	0.75 (0.70-1.60)	0.79 (0.67-4.2)	0.70 (0.70-1.60)	1.00
Platelet count,* x 10*^*9*^* per L*	216 (147-272)	180 (69-274)	223 (209-272)	0.25
Total bilirubin, *mg/dL*	0.77 (0.46-0.90)	0.80 (0.50-1.41)	0.47 (0.40-0.80)	0.13
**Lung and Kidney**				
Compliance _Rs,_ at baseline, *cm H*_2_*O*	26.5 (20-32)	27 (20-40)	25 (18-29)	0.40
Causes of lung injury				
Pneumonia, n 12(%) Sepsis, n 2(%) Trauma, n 0(%)	12 (85.7) 2 0	6 1 0	6 1 0	
KDIGO III, n (%)	14 (100.0)	7 (100.0)	7 (100.0)	-
Adjuvant therapy before ECCO_2_R				
Neuromuscular blockade Prone positioning Nitric oxide Recruitment maneuvers	14 (100.0) 6 (42.9) 0 14 (100.0)	7 (100.0) 2 (28.6) 0 14 (100.0)	7 (100.0) 4 (57.1) 0 14 (100.0)	

^a^*Quantitative variables are summarized as median and (IQR)*

*BMI* Body mass index, *SAPS* Simplified acute physiological score, *SOFA* Sequential organ failure assessment, *APACHE II score* (Acute Physiologic Assessment and Chronic Health Evaluation II), *PaO*_*2*_*/FiO*_*2*_ ratio of arterial-to-inspiratory oxygen fraction, *GCS* Glasgow coma score, Kidney Disease Improving Global Outcomes (KDIGO) class

Age, gender, Charlson Index, APACHE II, SAPS II and SOFA Score, underlying causes of lung injury and general clinical characteristics did not differ between the two groups. Respiratory failure adjuvant therapy pre-ECCO_2_R as recruiting maneuvers and prone positioning if applicable were performed [11, 12].

Overall clinical characteristics at different time-points: ventilatory settings, pH, blood gases, respiratory mechanics, coagulation and hemodynamics are reported in Table 2.

**Table 2:** Clinical characteristics at different time-points (*n*=14)

**Variables**^**a**^	**Baseline**	**1 hours**	**24 hours**	**48 hours**	**72 hours**	***p*-value**
Arterial pH	7.27 (7.22-7.31)	7.37 (7.31-7.39)	7.32 (7.26-7.37)	7.33 (7.31-7.36)	7.33 (7.29-7.36)	0.05
PaCO_2_ *mm Hg*	60.5 (56-77)	49 (44-58)	54.5 (47-61)	53.5 (46-61)	54 (47-61)	0.03^1^
PaO_2_ *mm Hg*	96 (79-121)	86.5 (81-96)	88 (82-106)	92 (81-97)	82 (73-92)	0.41
*PaO2/ FiO2 ratio*	146.5 (113-173)	114.5 (102-130)	115 (103-172)	134 (107-177)	132.5 (107-153)	0.22
Pplat *cm H*_2_*O*	30 (27-35)	25.5 (24-28)	27.5 (26-32)	27.5 (26-28)	27 (25-28)	0.001
V_T_ *ml/kg PBW*	6.7 (6.1-6.9)	5.1 (4.2-5.6)	5.2 (4.5-6.0)	5.0 (4.2-6.1)	5.2 (4.3-6.1)	<0.0001^2^
PEEP _estr_ *cm H*_2_*O*	11.5 (10-14)	14 (12-15)	13.5 (12-16)	14 (12-16)	14 (12-16)	0.03
PEEP _tot_ *cm H*_2_*O*	12.5 (10-15)	14 (12-15)	14 (13-16)	14.5 (13-16)	14 (13-16)	0.01
Driving pressure *cm H*_2_*O*	17 (14-18)	11.5 (10-15)	13.5 (11-17)	12.5 (11-17)	11.5 (11-14)	0.0001^3^
Compliance_Rs_ *cm H*_2_*O*	26.5 (20-32)	25 (18-36)	25.5 (15-33)	29 (16-36)	30 (22-36)	0.13
Heparin *IU/kg/h*	-	11 (10-13)	11 (11-13)	11.5 (10-14)	11 (10-14)	0.47
*a*PTTr sec	1.5 (1.2-1.8)	-	1.8 (1.5-2.1)	1.8 (1.5-2.0)	1.9 (1.8-2.0)	0.02
D-dimer µg/mL	2.4 (1.1-3.2)	-	1.3 (1.2-2.3)	1.7 (1.2-2.6)	2.0 (1.7-2.7)	0.97
*MAP, mm Hg*	70.5 (65-75)	70 (68-75)	68.5 (65-70)	72 (70-80)	75.5 (70-80)	0.08
Norepinephrine *μg/kg/min*	0.20 (0.06-0.52)	0.25 (0.10-0.55)	0.25 (0.10-0.60)	0.45 (0.10-0.70)	0.29 (0.08-0.50)	0.15
V_T_ *ml*	440 (380-450)	290 (280-360)	325 (280-350)	315 (280-380)	315 (280-400)	<0.0001^4^
Vent/min L*/min*	15.1 (12.2-15.8)	9.8 (9-11.5)	10.7 (8.9-12.3)	10.8 (7.8-13.3)	11.1 (8.4-14.0)	0.0001^5^

^a^Quantitative variables are summarized as median and (IQR)

Post-hoc comparison:

^1^Baseline VS. 1h *p*-value= 0.003

^2^Baseline VS. 1h *p*-value= 0.0001; baseline VS. 24h *p*-value= 0.0001; baseline VS. 48h *p*-value= 0.0004. baseline VS. 72h *p*-value= 0.0005

^3^Baseline VS. 1h *p*-value= 0.0004; baseline VS. 24h *p*-value= 0.001; baseline VS. 48h *p*-value= 0.003; baseline VS. 72h *p*-value= 0.0005

^4^Baseline VS. 1h *p*-value= 0.0001; baseline VS. 24h *p*-value= 0.0001; baseline VS. 48h *p*-value= 0.0005; baseline VS. 72h *p*-value= 0.0005

^5^Baseline VS. 1h *p*-value= 0.0009; baseline VS. 24h *p*-value= 0.003; baseline VS. 48h *p*-value= 0.004

*PaCO*_*2*_ Partial pressure of arterial CO_2_, *PaO*_*2*_ Partial pressure of arterial O_2_, *Pplat* Plateau pressure, *V*_*T*_ Tidal volume, *PEEP* Positive end-expiratory pressure (extrinsic or total); Driving pressure: driving pressure = Pplat minus PEEP; PaO_2_/FiO_2_ ratio: arterial-to-inspiratory oxygen fraction; *MAP* Mean arterial pressure

All patients who underwent ECCO_2_R+CRRT treatment presented significant reduction in V_T_ from baseline value of 6.7 ml/kg PBW (6.1-6.9) to 5.1 (4.2-5.6) after one hour, 5.0 (4.2-6.1) after 48 hours and 5.2 (4.3-6.1) after 72 hours (*p-value* <0.0001); the change in PaCO_2_ was from baseline 60.5 mmHg (56-77) to 49 (44-58) after one hour and 54 (47-61) after 72 hours (*p-value* 0.03). Enhancement in lung protection was followed by decrease in driving pressure from baseline 17 cm H_2_O (14-18) to 11.5 (10-15) after one hour and 11.5 (11-14) after 72 hours (*p-value* 0.0001) and P_plat_ from baseline 30 cm H_2_O (27-35) to 25.5 (24-28) after 1 hour and 27 (25-28) after 72 hours (*p-value* 0.001); all changes were not accompanied by significant effects on PaO2/FiO2 respiratory system compliance and hemodynamic status.

V_T_ reduction was coupled with PEEP increase avoiding conditions of derecruitment and alveolar opening-closing: from baseline values of 12.5 (10-15) cm H_2_O, PEEP was set to 14 (12-15) cm H_2_O after one hour and 14 (13-16) after 72 hours (*p-value* 0.01).

**Fig. 1:**
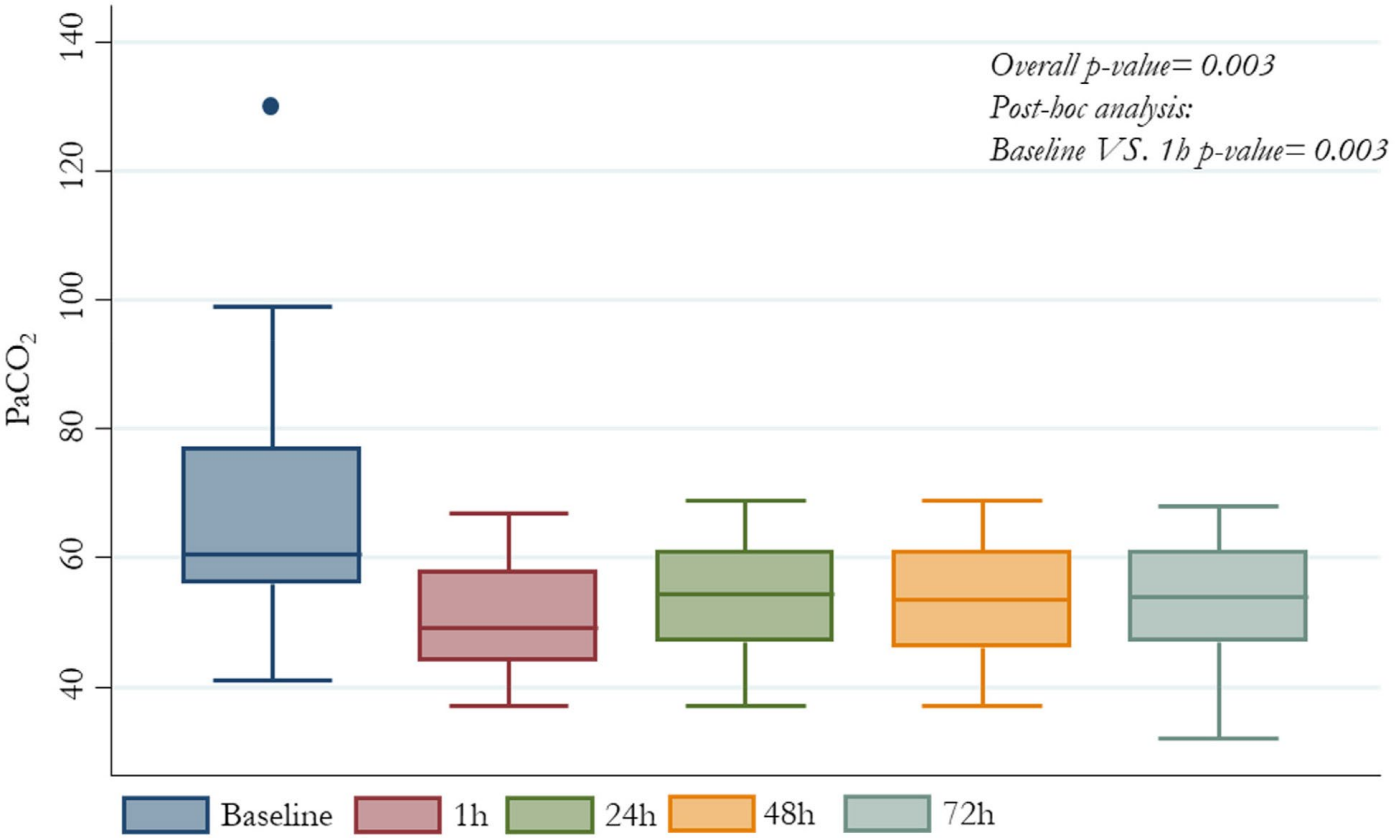
PaCO_2_ median values by different time-points

**Fig. 2:**
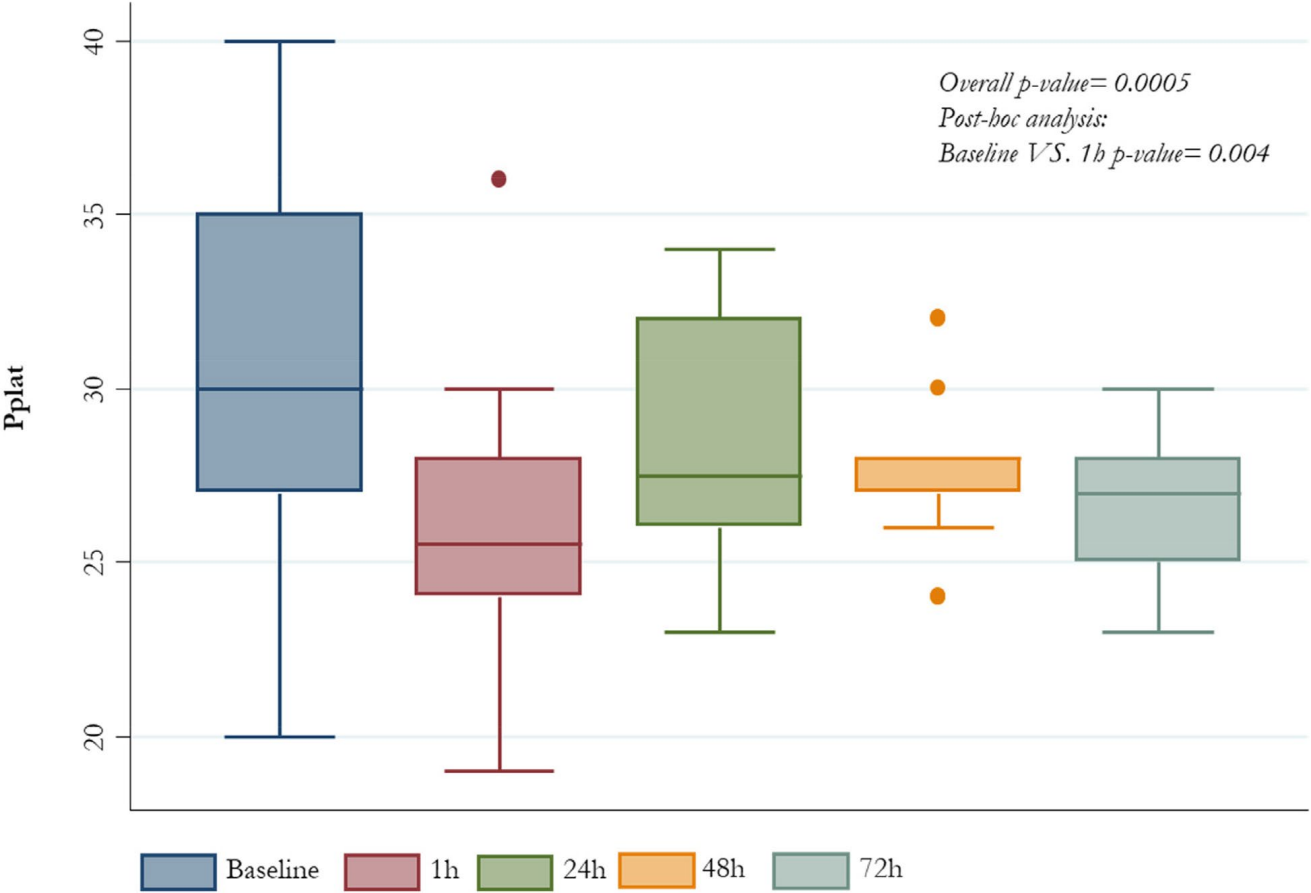
Pplat median values by different time-points

**Fig. 3:**
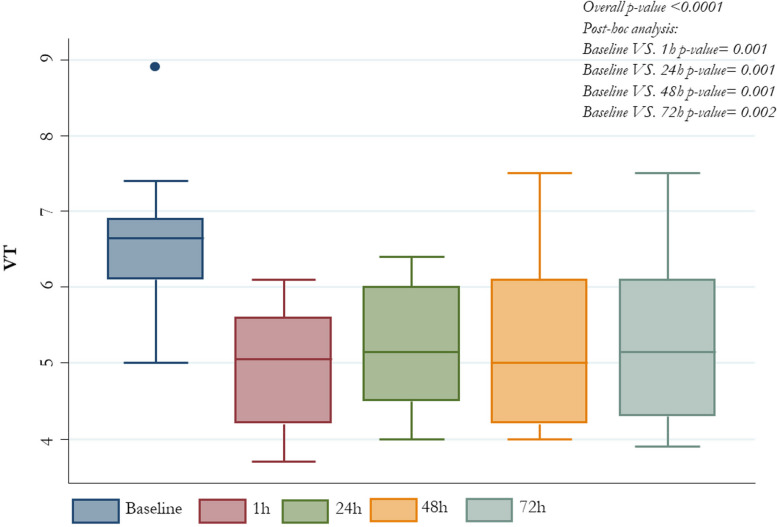
VT median values by different time-points

**Fig. 4:**
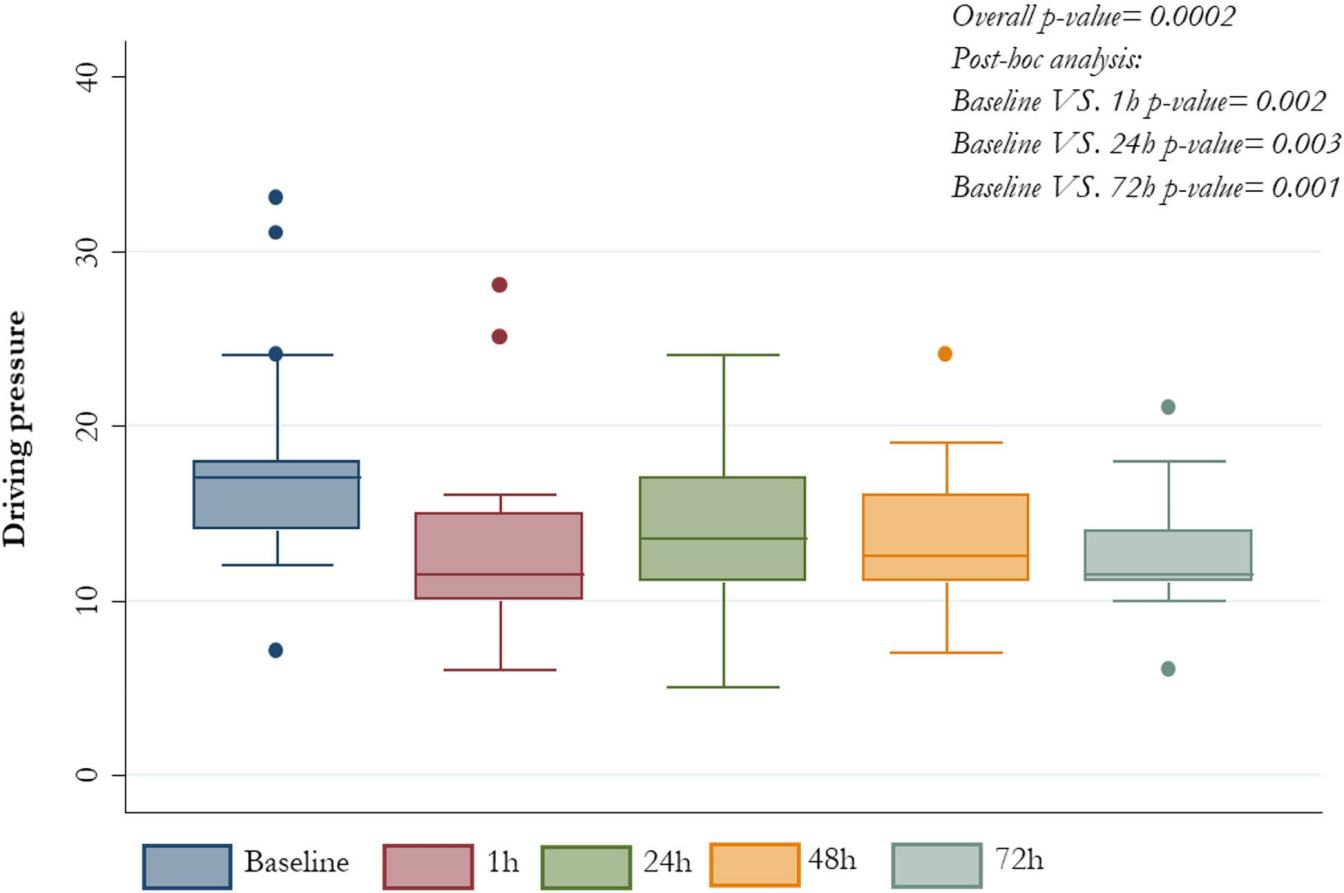
Driving pressure median values by different time-points

Overall driving pressure, PaCO_2_, P_plat_ and V_T_ patients’ values, at different time-points are also described in *Figures from *1* to *4, respectively (Figs. 1, 2, 3 and 4).

**Fig. 5:**
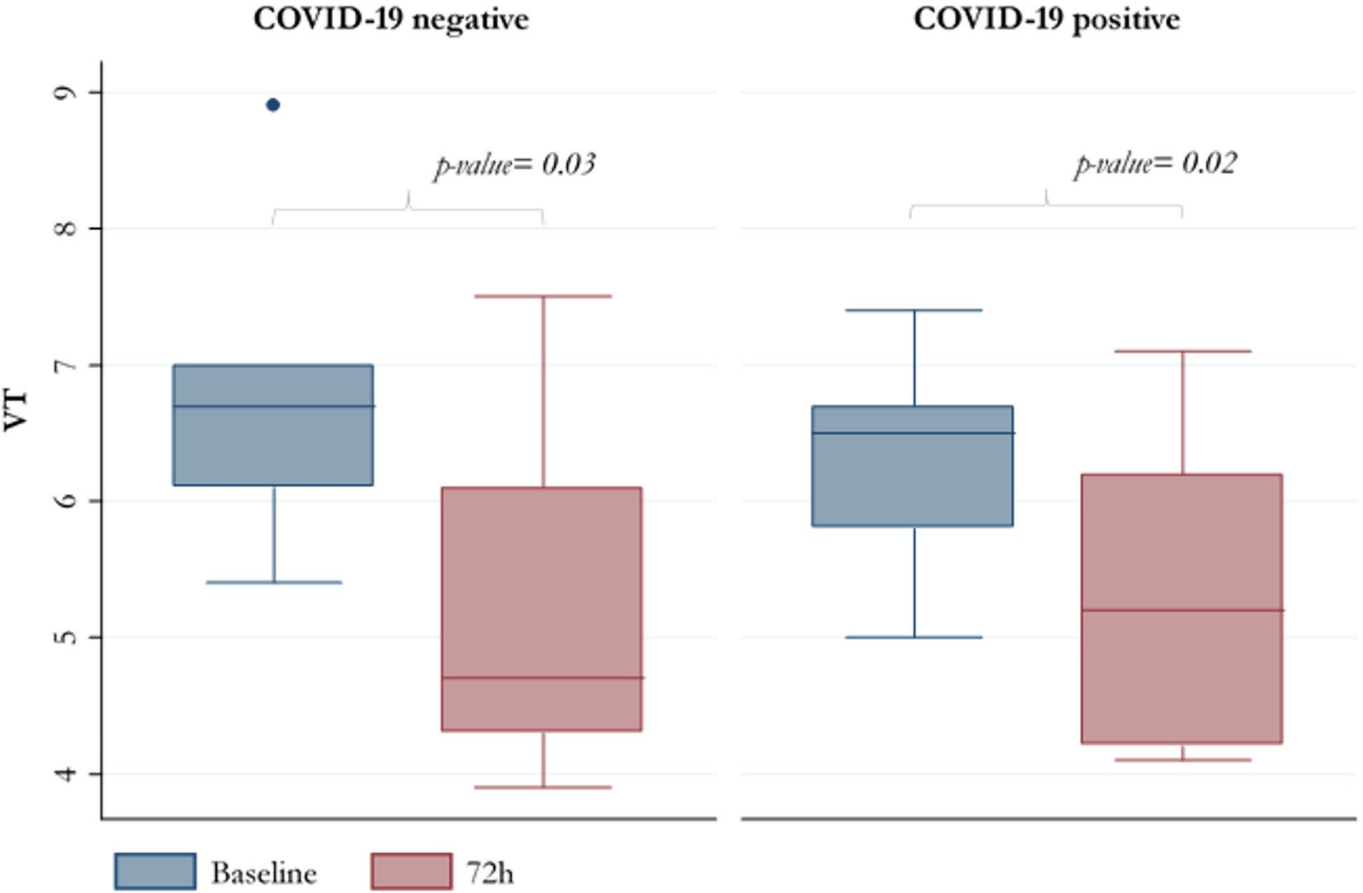
VT median values at baseline and at 72 hours

**Fig. 6:**
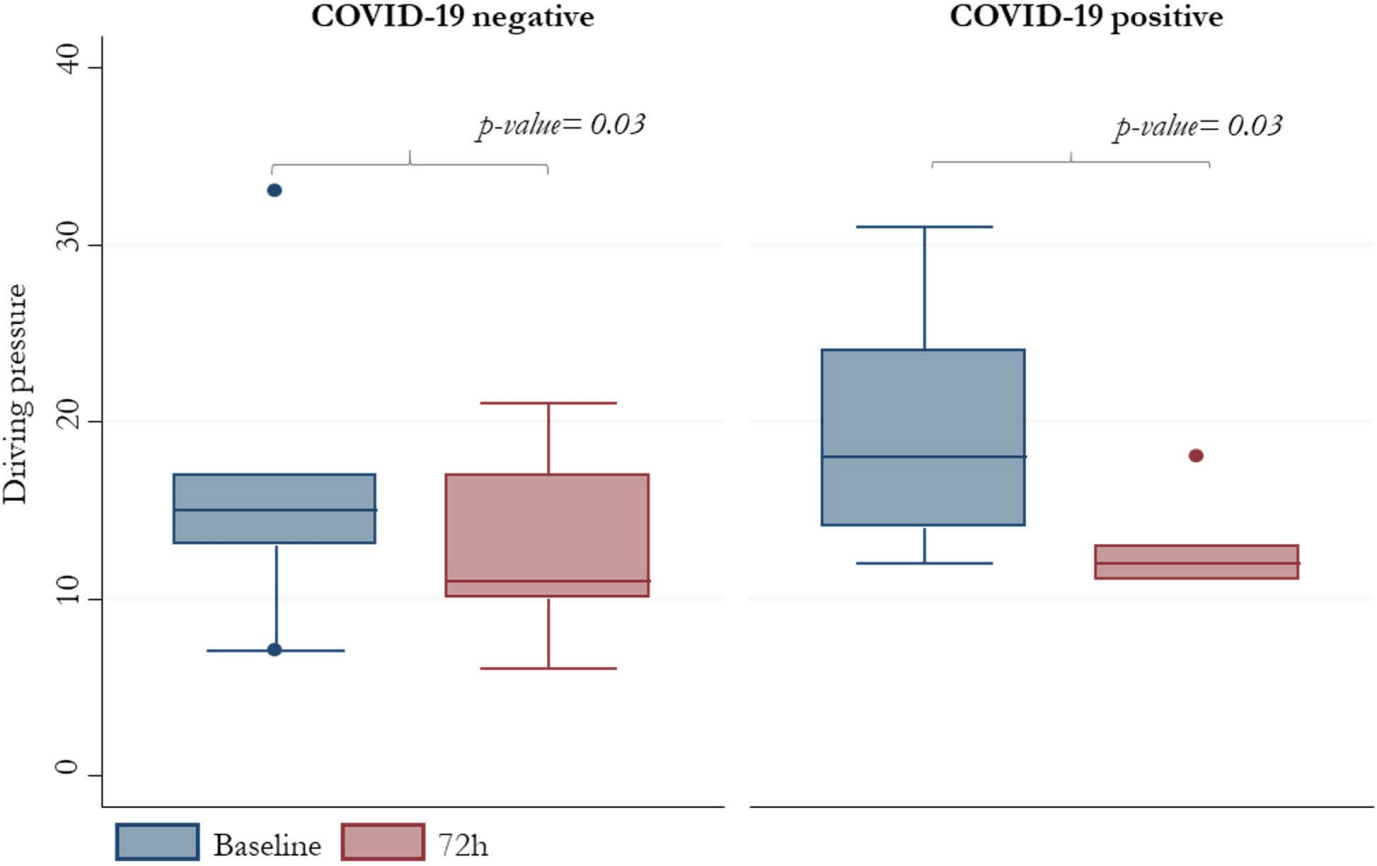
Driving pressure median values at baseline and at 72 hours

Driving pressure and V_T_ median values from baseline to 72 hours in the two groups (COVID negative and positive) are reported in Figs. 5* and *Fig. 6, respectively (Fig. 5, 6).

The decrease of driving pressure and V_T_ in the COVID-19 negative and positive patient groups from baseline to 72 hours was significant. Driving pressure decreased from 15 cm H_2_O (13-17) to 11 (10-17) after 72 hours (*p-value* 0.03) in the COVID-19 negative group and from 18 (14-24) to 12 (11-13) after 72 hours (*p-value* 0.004) in the COVID-19 positive group; V_T_ decreased in Covid-19 negative patients from 6.7 ml/kg PBW (6.1-7.0) to 4.7 (4.3-6.1) after 72 hours (*p-value* 0.003) and 6.5 (6.0-6.7) to 5.2 (4.2-6.2) after 72 hours (*p-value* 0.0005) in the COVID-19 positive group; (Tables 3 and 4).

**Table 3:** Clinical characteristics at different time-points stratified by COVID-19 patients

***Covid-19 NEGATIVE (n=7)***						
Variables*	**Baseline**	**1 hour**	**24 hours**	**48 hours**	**72 hours**	***p*-value**
Arterial pH	7.28 (7.24-7.32)	7.37 (7.30-7.43)	7.33 (7.29-7.39)	7.35 (7.32-7.47)	7.35 (7.31-7.42)	0.09
PaCO_2_ *mm Hg*	58 (54-72)	51 (44-56)	53 (47-64)	53 (47-61)	59 (53-61)	0.43
PaO_2_ *mm Hg*	119 (86-138)	87 (86-96)	106 (84-112)	95 (82-98)	81 (73-82)	0.08
*PaO2/ FiO2 ratio*	173 (140-225)	124 (107-201)	172 (116-235)	177 (131-204)	140 (130-202)	0.41
Pplat *cm H*_2_*O*	28 (22-30)	25 (22-30)	27 (24-32)	27 (24-30)	25 (24-29)	0.16
V_T_ *ml/kg PBW*	6.7 (6.1-7.0)	4.5 (4.0-5.7)	5.2 (4.0-6.1)	4.7 (4.0-6.1)	4.7 (4.3-6.1)	0.003
PEEP _estr_ *cm H*_2_*O*	11 (10-12)	12 (12-14)	12 (12-14)	13 (11-15)	13 (12-14)	0.16
PEEP _tot_ *cm H*_2_*O*	12 (10-13)	13 (12-14)	13 (13-14)	14 (12-16)	14 (13-15)	0.18
Driving pressure *cm H*_2_*O*	15 (13-17)	12 (10-16)	14 (10-19)	14 (10-17)	11 (10-17)	0.03
Compliance_Rs_ *cm H*_2_*O*	27 (20-40)	23 (18-36)	25 (13-43)	21 (15-42)	30 (14-43)	0.37
Heparin *IU/kg/h*	-	10 (10-11)	11 (10-11)	11 (10-15)	11 (8-15)	0.39
D-dimer µg/mL	2.5 (1.1-3.8)	-	1.3 (1.0-15.0)	1.2 (1.0-17.9)	1.8 (0.9-9.2)	0.60
*MAP, mm Hg*	70 (65-75)	70 (68-75)	68 (65-70)	72 (72-80)	80 (74-85)	0.03
Norepinephrine *μg/kg/min*	0.15 (0.06-0.50)	0.20 (0.08-0.50)	0.20 (0.08-0.60)	0.40 (0.08-0.70)	0.20 (0.06-0.30)	0.12
V_T_ *ml*	440 (380-460)	280 (250-320)	300 (280-350)	280 (280-420)	300 (270-420)	0.004
Vent/min L*/min*	12.2 (11.0-16.1)	9.0 (7.5-11.2)	9.8 (6.9-12.3)	9.8 (5.0-14.7)	10.5 (5.6-14.7)	0.06

^*a*^*Quantitative variables were summarized as median and (IQR)*

*PaCO*_*2*_ Partial pressure of arterial CO_2_, *PaO*_*2*_ Partial pressure of arterial O_2_, *Pplat* Plateau pressure, *V*_*T*_ Tidal volume, *PEEP* Positive end-expiratory pressure (extrinsic or total); Driving pressure: driving pressure = Pplat minus PEEP; PaO_2_/FiO_2_ ratio: arterial-to-inspiratory oxygen fraction; *MAP* Mean arterial pressure

**Table 4:** Clinical characteristics at different time-points stratified by COVID-19 patients

***Covid-19 POSITIVE (n=7)***						
**Variables**^**a**^	**Baseline**	**1 hour**	**24 hours**	**48 hours**	**72 hours**	***p*-value**
Arterial pH	7.24 (7.22-7.31)	7.37 (7.34-7.38)	7.26 (7.24-7.35)	7.32 (7.20-7.35)	7.29 (7.27-7.35)	0.24
PaCO_2_ *mm Hg*	67 (57-85)	47 (43-61)	55 (44-61)	54 (44-65)	54 (44-66)	0.06
PaO_2_ *mm Hg*	85 (79-117)	85 (78-96)	84 (72-91)	82 (69-95)	92 (72-107)	0.77
*PaO2/ FiO2 ratio*	113 (107-156)	106 (87-128)	103 (87-114)	131 (72-137)	113 (72-141)	0.57
Pplat *cm H*_2_*O*	34 (30-35)	26 (24-28)	28 (27-32)	28 (27-28)	27 (26-28)	0.006
V_T_* ml/kg PBW*	6.5 (6.0-6.7)	5.2 (4.2-5.6)	5.1 (4.5-5.6)	5.1 (4.2-6.2)	5.2 (4.2-6.2)	0.0005
PEEP _estr_ *cm H*_2_*O*	14 (10-15)	14 (11-15)	15 (10-16)	15 (12-16)	15 (12-16)	0.27
PEEP _tot_ *cm H*_2_*O*	14 (11-16)	15 (11-15)	16 (12-17)	16 (13-17)	16 (13-17)	0.04
Driving pressure *cm H*_2_*O*	18 (14-24)	11 (10-15)	12 (11-17)	12 (11-17)	12 (11-13)	0.004
Compliance_Rs_ *cm H*_2_*O*	25 (18-29)	27 (17-40)	29 (15-33)	32 (16-35)	32 (22-36)	0.37
Heparin *IU/kg/h*	-	13 (11-13)	13 (11-14)	12 (10-14)	11 (10-12)	0.36
D-dimer µg/mL	2.4 (1.2-2.7)	-	1.3 (1.2-2.3)	1.9 (1.6-2.6)	2.4 (2.0-2.7)	0.32
*MAP, mm Hg*	71 (65-75)	70 (65-70)	70 (65-75)	70 (65-84)	75 (68-77)	0.85
Norepinephrine *μg/kg/min*	0.30 (0.00-0.60)	0.30 (0.10-0.60)	0.35 (0.10-0.70)	0.50 (0.10-0.70)	0.50 (0.20-0.70)	0.08
V_T_ *ml*	440 (370-450)	300 (280-400)	330 (290-360)	342 (280-380)	356 (280-400)	0.0005
Vent/min L*/min*	15.4 (13.0-15.8)	10.5 (9.2-14.0)	11.2 (8.9-12.6)	11.2 (7.8-13.3)	11.5 (8.4-14.0)	0.001

^a^Quantitative variables were summarized as median and (IQR)

*PaCO*_*2*_ Partial pressure of arterial CO_2_, *PaO*_*2*_ Partial pressure of arterial O_2_, *Pplat* Plateau pressure, *V*_*T*_ tidal volume, *PEEP* Positive end-expiratory pressure (extrinsic or total); Driving pressure: driving pressure = Pplat minus PEEP; PaO2/FiO2 ratio: arterial-to-inspiratory oxygen fraction; *MAP* Mean arterial pressure

## ECCO_2_-R treatment

Blood-flow rates from 270 ml/min to 430 ml (mean 365 ml/min at 24 hours) were achieved for all patients during ECCO_2_R+CVVHD treatment. No patient-related complications were observed. Mechanical complications during extracorporeal procedure time (median 96 {63-120} hours) were recorded as: two cases of membrane lung/hemofilter clotting (only in the COVID-19 affected group) and one case of pump malfunction. Mean heparin doses were: 11 IU/kg/h (10-13) and 11 IU/kg/h (10-14) at the start and at the end of the treatment, respectively; while it was observed an *a*PTT*ratio* median value of 1.8 (1.5-2.1) and 1.9 (1.8-2.0) at 24 and 72 hours of treatment, respectively. Technologies to remove CO_2_ applied to our patients were not equipped with systems to automatically measure CO2 clearance by ECCO_2_R. The amount of CO_2_ removed by the oxygenator was measured through tests based on clinician requests using standard formulas [13]. In three patients, the CO_2_ cleaned by the artificial lung (ml/min) was calculated as the partial pressure of CO_2_ of gases exiting the artificial lung (mmHg, measured by the capnograph, monitor IntelliVue MX750 with Microstream CO_2_ Extension^®^, Philips Medical Systems) divided by 713 mmHg, and multiplied by the gas flow value (ml/min).

During the first 72 hours of extracorporeal treatment, analyses showed a constant CO_2_ clearance with a mean value of 101 ml/min (SD 11 ml/min) useful for V_T_ reduction coupled in one case also to the respiratory rate limitation.

Overall patient’s ECCO_2_-R duration was 96 (63-120) hours and 96 (72-225) or 63 (24-120) in COVID-19 negative or positive groups (*p-value* 0.37), respectively.

## Outcome of patients

No statistically significant differences were recorded on treatment effects between patients with moderate ARDS with COVID disease and the control group in the pre-pandemic period. MV, ICU length of stay (LOS) and Day-28 mortality, are reported in Table 5.

**Table 5:** Patients’ outcome

**Variables**^**a**^	**All sample** **(*n*= 14)**	**COVID-19 negative (*n*= 7)**	**COVID-19 positive (*n*= 7)**	***p*-value**
*Mechanical ventilation (days)*	23.5 (7-33)	24 (22-38)	19 (7-29)	0.24
*ICU length of stay (days)s*^*a*^	23.5 (7-36)	24 (22-78)	19 (7-29)	0.25
*Day-28 mortality, n (%)*	8 (57.1)	4 (57.1)	4 (57.1)	1.00
**SOFA parameters at the end of ECCO**_**2**_**R**				
*SOFA total score*	8.5 (8-11)	9 (8-9)	8 (6-12)	0.92
*GCS*	14 (14-15)	14 (7-14)	15 (14-15)	0.08
*PaO*_*2*_*/FiO*_*2*_* ratio*	133 (100-242)	242 (132-338)	102 (57-141)	0.04
*Mean Arterial Pressure, mm Hg*	70.5 (68-75)	70 (68-75)	71 (65-75)	0.76
*Creatine kinase, mg/dL*	1.09 (0.66-1.59)	0.92 (0.68-1.36)	1.26 (0.51-1.62)	0.83
*Platelet count, x 10*^*9*^* per L*	196 (130-320)	133 (106-320)	315 (188-329)	0.25
*Total bilirubin, mg/dL*	0.70 (0.50-1.30)	0.70 (0.60-1.00)	0.50 (0.40-3.50)	0.59

^a^Quantitative variables are summarized as median and (IQR)

*ICU* Intensive care unit, *ECCO*_*2*_*R* Extracorporeal carbon-dioxide removal, *SOFA* sequential organ failure assessment, *PaO*_*2*_*/FiO*_*2*_ Ratio of arterial-to-inspiratory oxygen fraction, *GCS* Glasgow coma score

Significant increase in PaO_2_/FiO_2_ *ratio* was detected in COVID-19 negative patients (242 {132-338} *vs* 102 {57-141} *p-value* 0.04). No other significant differences in SOFA score at the end of ECCO_2-_R treatment were recorded. LOS in ICU (23.5 days {7-36} and day-28 mortality {*n* = 8 (57.1%)} did not differ in the two groups.

## Discussion

Severe critical illnesses, such as acute lung injury with acute respiratory distress syndrome as more severe subset, as well as the need of CRRT to bridge organ dysfunction is a common experience for clinicians in ICUs to contrast increased mortality and morbidity; a similar scenario also occurred during the Covid-19 pandemic emergency.

The concept “positive pressure MV can save lives” was proved in the 1950s during the poliomyelitis epidemics, but the following development of critical care medicine demonstrated that ventilatory treatment could cause a form of injury known as ventilator induced lung injury (VILI) that is clinically indistinguishable from ARDS [1, 14, 15].

ARDS accounts for 10% of ICU admissions, and the overall ICU mortality ranges from 35% for mild ARDS to 46% for severe ARDS [16].

Significant progress has been made in understanding the pathophysiology of this syndrome and the ability to recognize VILI has led to radical modifications of the ventilatory management with new ventilator modes and settings.

Several studies demonstrated that the main reason for high mortality (30%-50%) is not the severe hypoxemia but rather the multi-organ failure (kidney, heart, liver, etc.), potentially caused by inflammatory mediators due to and/or augmented by artificial ventilation that are delivered from the lungs through the systemic circulation to peripheral organs. The use of high tidal volumes and high airway pressures has been shown to be deleterious for patient outcomes, and thus protective ventilation strategies, including lower tidal volumes and the new ultra-low volume protective MV, have been implemented into clinical practice [3, 4, 17, 18].

In the past two decades studies have shown that lung hyperinflation still occurs in approximately 30% of ARDS patients even though they are being “correctly” ventilated using the ARDS*Net* strategy [19]. These investigations also suggested that some patients could benefit from a further reduction of V_T_ even when P_plat_ is lower than 30 cmH_2_O [3, 20]. Bellani and coworkers assessed the intensity of pulmonary inflammation during MV using positron-emission tomography imaging to detect the presence of metabolically active inflammatory cells. They showed that P_plat_ is significantly correlated with metabolic activity and this phenomenon sharply increases sharply above 26-27 cmH_2_O, thus suggesting that further limitation of ventilation to values of 25 cmH_2_O or lower may be associated with lower degree of pulmonary inflammation due to reduced VILI [8].

However, lower tidal volumes in clinical practice have been proved successful but can be extremely problematic when dealing with respiratory acidosis. The implementation of extracorporeal CO_2_ removal technique can represent the missing link between prevention of VILI and pH control [4, 21, 22].

From the first pioneering ECCO_2_-R [23], the new treatments derived from ECMO support have seen increased interest being characterized by less invasive approaches with low flow devices allowing an easier management of ARDS patients to reach the ultra-protective ventilation characterized by V_T_ < 6 mL/kg PBW and P_plat_ ≤ 25 cmH2O [4].

In the multicenter SUPERNOVA study, sponsored by the European Society of Intensive Care Medicine, Combes et al. tried to assess safety and feasibility of ECCO_2_-R in ARDS [24].

More than 80% of patients with moderate ARDS might achieve ultraprotective ventilation goals by using ECCO_2_-R. In the study design authors used lower and higher CO_2_ extraction devices (membrane lung cross-sectional area 0.59 vs. 1.30 m^2^; flow 300–500 mL/min vs. 800–1000 mL/min, respectively).

Efficacy and safety were higher for devices with higher blood flow and use of extracorporeal support allowed a reduction in V_T_ from approximately 6 to 4 mL/kg PBW with significant decrease in driving pressure from 13 to 9 cmH_2_O.

The main result of the present study was a successful achievement of the targeted driving pressure (≤ 14 cmH_2_O) in all patients within the first 24 hours and maintained after 72 hours. Specifically in the group of patients with ARDS associated with COVID-19 the achievement of the targeted driving pressure and the protective mechanical ventilation was more effective and achieved in a shorter time if compared to the historical control group where ARDS was not associated with COVID-19; as a matter of fact, in those patients the driving pressure at baseline was more severe (18 {14-24} cmH_2_O) compared to the historical group (15 {13-17} cmH_2_O) and the reduction after the first 24 hours was greater (11 {10-15} cmH_2_O vs 12 {10-16} cmH_2_O). In the SUPERNOVA study, the authors showed that only 64% of the patients treated with a low flow system achieved an ultra-protective mechanical ventilation with a Vt<4 ml/kg*PBW and a Pplat < 25 cmH_2_O, compared to 92% of those who received a high flow system. Although, in our study we did not reach a Vt<4 ml/kg*PBW and a Pplat < 25 cmH_2_O in the majority of our patients, especially in those with COVID-19, we showed that through a low flow device all patients achieved a protective ventilation strategy with a significant reduction of the driving pressure ≤ 14 cmH_2_O, from 17 (14-18) to11.5 (11-14) cmH_2_O within the first 24 hours. However, the use of high flow devices in the SUPERNOVA trial allowed significant reductions in RR that our study was able to achieve only in one case. RR reduction is the limit of low flow ECCO_2_-R systems; a more protective ventilatory setting can only be achieved by increasing the flow.

Although the treatment did not affect 28 days mortality, duration of mechanical ventilation and ICU length of stay were higher in patients from the historical group of ARDS compared to COVID-19.

Acute kidney injury (AKI) afflicts a large number of ICU patients and carries high morbidity and mortality. In this scenario, ARDS patients with AKI have been shown to present significantly higher mortality than patients without AKI in several cohorts; AKI may develop in 25%–60% of ARDS patients, commonly when sepsis is the underlying disease [5, 6].

According to the ARDS*net* trial data base, among all participants enrolled who did not have end-stage renal disease, 24% developed AKI over the first four study days [5, 19]. In this context, MV is an independent risk factor for mortality in patients with AKI; likewise, the increase in plasma concentrations of inflammatory mediators and apoptosis of renal tubular cells are also associated with AKI [25]. Starting from these assumptions, recent studies have proposed the inclusion of ECCO_2_-R into the conventional CRRT circuit to support lung and kidney functions simultaneously [26–29].

A recent trial on ARDS patients demonstrated efficacy when applying CRRT plus ECCO_2_-R with ultra-protective ventilation to preserve renal function through attenuation of inflammation and apoptosis [7].

Since December 2019, the outbreak of coronavirus disease caused by SARS-CoV-2, has become one of the main causes of ARDS. Although both ARDS and COVID-19 lung injury present with several pathophysiological features such as gas exchange impairment, alveolar flooding and vasculopathy, it has been showed that COVID-19 lung injury involves direct viral damage and a host defense response with greater vasculopathy (macro- and micro thrombosis), endothelial cell injury, vascular dilation, aberrant angiogenesis and inflammatory reactions with a particular tropism for cells where ACE2 receptor is expressed (lung, brain, heart, intestines, liver and kidney) [30]. In COVID-19 patients, mainly for those who received MV, mortality ranged from 13% to 69% [31].

The mechanisms of AKI in COVID-19 could be multifactorial, due to cytokine damage, cardio-renal crosstalk, hypoxia, intra-abdominal hypertension, fluid imbalance, hypoperfusion, rhabdomyolysis-related tubular toxicity and endotoxin. Large studies on clinical characteristics of patients affected by coronavirus disease 2019 in China reported that prevalence of AKI was only 0.5% but severe cases showed higher percentage of AKI (23%) [32, 33].

Our study updates the new concepts on ultra-protective ventilation with the use of low flow CO_2_-Removal technique supported by CRRT both in standard ARDS with AKI during the pre-pandemic era as well as over the past three years, characterized by the known COVID-19 infection disease.

In patients where both acute lung damage and AKI coexist, an oxygenator to remove CO_2_ could be implemented with an hemofilter for CRRT, placed in series upstream or downstream position, combining both CO_2_ removal and hemodiafiltration technique for renal support.

However, data on these combined techniques are poor due to their complexity and due to the specific prerogative of some referral centers and narrowness of indications, although during the last pandemic emergency an increased number of critically ill patients with SARS-CoV-2 related pneumonia and extra pulmonary complications, including AKI, has been observed.

Therefore, our experience is here reported for the pre-pandemic period and over the past three years on COVID-19 patients with ARDS and AKI treated with a combination of CRRT and ECCO_2_R.

Even if limited to our restricted sample size, the reported data by comparing the presence and absence of the COVID pathology might be useful to bring out the applicability of the technique and its effectiveness in reducing CO_2_ levels to achieve less invasive and higher MV protection.

The new available technologies with integrated platform have given comparable results in terms of efficacy in CO_2_ removal, pH control, reduction of lung volumes and ventilation pressures with adequate level of renal support; furthermore, the efficacy of extracorporeal treatments in our patients were constant over time (the membrane maximum duration was manufacturer-determined to reach 72 hours).

In the SUPERNOVA study, authors demonstrated notable results with low incidence of adverse events (2%) [24]. In our study, despite the presence of coagulation disorders in the group of COVID-19 patients and the need of heparin treatment, we observed few side effects (i.e., two cases of circuit clotting and one case of pump malfunction). In addition, the combination of CRRT+ECCO_2_R was tested, changing the circuit, when necessary, over a 72-hour period in patients who failed the weaning from extracorporeal lung support.

Reasoning should be spent on one case, in which ECCO_2_R allowed an effective decrease of RR and ventilation flow.

**Fig. 7:**
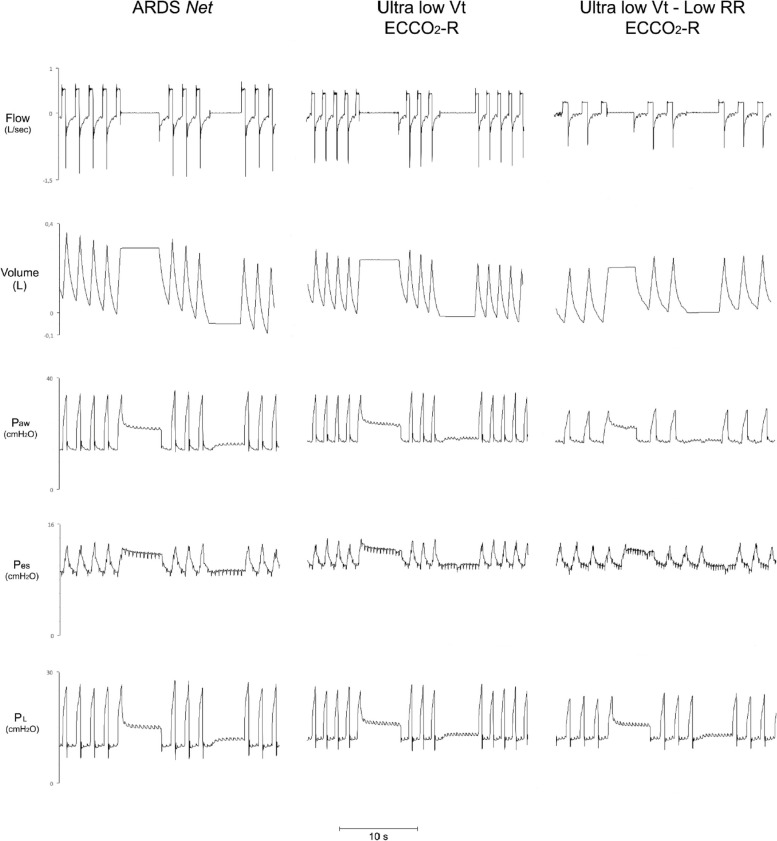
Exemplary time courses for clinical effects of low-flow CO2 removal in patient with moderate ARDS due to pneumonia. A favorable response to ECCO_2_R treatment with sequential option for RR reduction. Figures represent three conditions: the first shows a standard treatment with ARDS *net* protocol application; the second shows an ultra-protective strategy (Ultra low Vt and high RR) supported by ECCO_2_R; the third illustrates a strategy coupling low Vt and low RR with ECCO_2_R support. In the two cases supported by extracorporeal support the values of PEEP were increased to reduce conditions of opening-closing avoiding hyperinflation. From top to bottom: tracings of flow, volume, airway, esophageal and transpulmonary pressure. Paw = airway pressure; Pes = esophageal pressure; PL = transpulmonary pressure

Figure 7 shows exemplary time courses for clinical effects of low-flow CO_2_ removal in a patient with moderate ARDS due to pneumonia presenting favorable response to treatment with ECCO_2_-R with sequential option for RR and flow reduction.

One of the first studies on correlation between RR and lung injury was proposed by Hotchkiss, et al. in an animal model where the decrease in RR enabled to diminish the severity of VILI through lessening edema formation and perivascular hemorrhage [34]. According to the authors, although drawing parallels between the experimental setting and clinical practice was clearly hazardous, it was conceivable that in the early ARDS phase, similar mechanisms might occur.

Recently, Grasso et al., demonstrated on an experimental ARDS model that a low RR plus ECCO_2_R decrease systemic and pulmonary inflammatory mediators [35].

In the latter study, new concepts on pulmonary protection suggest considering other components of mechanical power contributing to negative or positive effects on lung mechanics, such as the RR or the flow to generate low V_T_. On this topic Rich et al. demonstrated that decreasing RR mitigates VILI only if the inspiratory flow rate decreases at the same time [36].

The continuous technological evolution with less invasive and more biocompatible extracorporeal circuits alongside with the new small artificial lungs increasingly performing with constant functions in CO_2_ clearance over time are changing our approach to extracorporeal treatments in moderate ARDS patients. Therefore, the opportunity in terms of lung protection to couple different elements such as low V_T_, driving pressure, P_plat_ and low RR in few cases (as for the exemplary time course reported) might provide clinicians with new strategies based on physiological evidence derived by research on animal models that once translated to pathological conditions might clarify the rationale behind its clinical application.

Our study has some limitations. The population enrolled was small and limited to patients who presented with moderate ARDS condition (even if PaO2/FiO2 ratio was less than 150 mm Hg) during the early phase of the renal disease requiring CRRT initiation. Given that the sample was not representative of specific stages of renal disease, extrapolating results to compare to other conditions requires caution.

Although our ICU represents the regional reference center for respiratory failure, the specific population enrolled is limited; thus, we had to extend the analysis to a three-year period to reach two comparable patient samples for the pre- Covid 19 period and for the Covid-19 pandemic emergency period.

## Conclusions

In moderate ARDS patients with or without COVID-19 disease presenting with respiratory acidosis during protective MV, ECCO_2_R+CRRT may be an effective supportive treatment to reach protective values of driving pressure unless severe oxygenation defects arise requiring ECMO therapy initiation.

Although the physiological bases of CO_2_ removal have been translated to pathological conditions, a reappraisal of the effects of ECCO_2_R and the potential crosstalk between lung and kidney might help clinicians to clarify the rationale behind its clinical application.

Further evidence from randomized clinical trials and/or high-quality prospective studies are warranted to better guide clinicians during the decision-making process.

## Consent for publication

We confirm that this manuscript has not been published elsewhere and Is not under consideration by another journal.

## Competing interests

All the other authors declare no competing interests.

## Data Availability

The datasets used and analyzed during the current study are available from the corresponding author on reasonable request; we anonymized the patients’ identities on the system and saved whole data in Excel form.

## Abbreviations

ECCO_2_R: Extracorporeal CO2 Removal
SARS-CoV-2: Severe acute respiratory syndrome coronavirus 2
COVID-19: Coronavirus disease-19
MV: Mechanical ventilation
VILI: Ventilator Induced Lung Injury
BMI: Body mass index
PBW: Predictive body weight
V_T_: Tidal Volume
ECMO: Extracorporeal membrane oxygenation
PEEP: Positive end-expiratory pressure
Pplat: Plateau pressure
RR: Respiratory rate
AKI: Acute kidney injury
KDIGO: Kidney diseases: improving global outcomes
CRRTHDF: Continuous Renal Replacement Therapy hemodiafiltration
CRRT: Continuous Renal Replacement Therapies
CRRTHDF: Continuous Renal Replacement Therapy hemodiafiltration
SOFA: Sequential Organ Failure Assessment
APACHE II: Simplified Acute Physiology II
ARF: Acute respiratory failure
IQR: Interquartile range
ICU: Intensive care unit
LOS: Length of stay
